# Unique Nerve Tissue‐Restricted T‐Cell Clones in Chronic Inflammatory Demyelinating Polyneuropathy

**DOI:** 10.1111/jns.70006

**Published:** 2025-02-18

**Authors:** G. G. A. van Lieverloo, D. C. Anang, M. E. Adrichem, B. A. Coert, A. E. Aronica, L. Wieske, I. N. van Schaik, N. de Vries, F. Eftimov

**Affiliations:** ^1^ Amsterdam Rheumatology and Immunology Center Amsterdam, Department of Clinical Immunology and Rheumatology, Amsterdam UMC University of Amsterdam Amsterdam the Netherlands; ^2^ Department of Neurology, Amsterdam UMC University of Amsterdam Amsterdam the Netherlands; ^3^ Department of Genome Analysis, Amsterdam UMC University of Amsterdam Amsterdam the Netherlands; ^4^ Department of Neurology Spaarne Gasthuis Haarlem the Netherlands; ^5^ Department of Neurosurgery, Amsterdam UMC University of Amsterdam Amsterdam the Netherlands; ^6^ Department of Neuropathology, Amsterdam UMC University of Amsterdam Amsterdam the Netherlands; ^7^ Department of Clinical Neurophysiology Sint Antonius Hospital Nieuwegein the Netherlands; ^8^ Sanquin Blood Supply Foundation Amsterdam the Netherlands

**Keywords:** CIDP, clonality, nerve, next‐generation sequencing, T‐cell receptor repertoire

## Abstract

**Background and Aims:**

Chronic inflammatory demyelinating polyneuropathy (CIDP) is an immune‐mediated disorder characterized by peripheral nerve damage. Although T lymphocytes (T‐cells) are implicated in the pathogenesis of CIDP, we previously observed that the frequency of highly expanded T‐cell clones (HECs) in peripheral blood of CIDP patients was not different from healthy controls. To investigate if local T‐cells might be pathogenic, we employed next‐generation sequencing to compare the TCRβ repertoire between peripheral blood and nerve tissue of CIDP patients.

**Methods:**

Adaptive immune receptor repertoire sequencing (AIRR‐Seq) of the TCRβ chain was conducted on peripheral blood and nerve tissue obtained from three newly diagnosed CIDP patients.

**Results:**

All patients showed high numbers of highly expanded TCRβ clones in nerve tissue that were not detected or detected only in very low frequencies in blood, whereas in blood other HECs were found. Clustering analysis based on CDR3‐similarity showed that these nerve tissue‐restricted TCRβ clones were distinct from blood clones, as evidenced by the absence of prominent clusters.

**Interpretation:**

Unique nerve tissue‐restricted TCRβ clones may indicate a highly localized immune response with localized expansion and/or retention of T‐cells that could contribute to the pathomechanism of CIDP.

Further characterization of the phenotype, antigen target and functionality of these T‐cells is essential to determine their pathogenic role.

## 1 Introduction

Chronic inflammatory demyelinating polyradiculoneuropathy (CIDP) is an immune‐mediated disorder characterized by inflammation of the peripheral nerve myelin sheath. Previous studies have highlighted the involvement of T‐lymphocytes in CIDP pathology, identifying oligoclonal expansions of CD8+ T‐cells in both peripheral blood and nerve biopsies using CDR3 spectratyping [1, 2]. However, recent findings from our research group, using high‐throughput next‐generation sequencing (NGS) in a large CIDP cohort, revealed stable frequencies of highly expanded T‐cell clones (HECs) in peripheral blood samples of both healthy controls and CIDP patients in different disease phases. This suggests that peripheral blood might not be the right compartment to study T‐cell mediated autoimmunity in CIDP [3]. The contrasting results between our study and the previous ones may be explained by the less sensitive and less specific CDR3 spectratyping methods used in those studies to assess changes in the TCR repertoire [1, 2], whereas NGS allows for more accurate and in‐depth clonal analyses, enabling the detection of low‐frequency clones.

The breakdown of the blood‐nerve barrier is considered a key event in CIDP pathogenesis, allowing circulating lymphocyte infiltration into nerve tissue despite its normally impermeable nature [3]. Whether the blood compartment accurately reflects T‐cell mediated pathology at the tissue level remains uncertain. Evidence of T‐cell infiltration in nerve biopsies of CIDP patients suggests that antigen‐specific T‐cells may preferentially migrate into nerve tissue, contributing to nerve damage. To explore this hypothesis, we conducted next‐generation sequencing to analyze the TCRβ repertoire in paired nerve tissue and peripheral blood samples obtained from three CIDP patients at the time of CIDP diagnosis.

## 2 Material and Methods

### 2.1 Patients and Sample Collection

In a prospective study, we included three patients suspected to have a CIDP variant [4, 5]. Nerve biopsies of the superficial peroneal nerve were performed for diagnostic purposes. Nerve biopsies are not a standard procedure in the diagnosis of CIDP and are only reserved for cases where diagnostic uncertainty persists, especially when the criteria for demyelinating nerve conduction are not fully met. Leftover biopsy material was utilized for this study. At the time of the nerve biopsy, blood sampling was performed as well. Nerves were considered suitable for biopsy if they were clinically affected, demonstrated some nerve conduction excitability, and/or focal enlargement on nerve ultrasound. The study protocol was approved by the local Medical Ethical Committee, and participants provided informed consent before inclusion.

Peripheral blood samples were collected in Paxgene tubes (2.5 mL) for RNA isolation, and serum isolated following a standardized protocol. Nerve biopsies were immediately preserved in RNAlater reagent (Qiagen), then stored at −80°C until analysis.

### 2.2 RNA Isolation, Next Generation Sequencing and Bioinformatics Analysis

The protocol for RNA isolation, cDNA synthesis, linear amplification and next generation sequencing has been previously described in detail [6, 7, 8]. In short, a linear amplification of the TCRβ repertoire was performed using primers covering all functional *V*_beta_ and *C*_beta_ genes. Samples were then purified, quantified, prepared for sequencing according to the manual for amplicon sequencing, and sequenced on a Roche Genome Sequencer FLX (titanium platform) or Illumina MiSeq platform. The obtained sequencing reads were analyzed with an in‐house developed pipeline for repertoire analysis “RESEDA” (REpertoire SEquencing Data Analysis, https://bitbucket.org/barbera/reseda), using the following steps: (1) pairwise assembly of the paired‐end reads using PEAR [9], (2) identification of the 8 bp MID, (3) identification of the complement determining region 3 (CDR3), (4) alignment to the IMGT database [10] to obtain the Variable and Joining gene assignment, (5) removal of reads with low quality bases (Q score < 30) in the CDR3, (6) clustering of reads in clones based on V gene name, J gene name and 100% amino acidic CDR3 identity, (7) contamination check between samples from different individuals. The final list of clones obtained from the RESEDA pipeline were analyzed with in‐house developed R scripts using R version 4.1 [11] using R studio. Based on previous studies in blood, TCRβ clones with a frequency ≥ 0.5% of the total TCRβ repertoire are referred to as highly expanded clones (HECs). There are no cut‐off values for TCRβ clones in nerve tissue, since normally T‐cells do not reside in nerve tissue. The impact of a set of clones is the sum of the frequencies of these clones.

### 2.3 Clustering Analysis

Clones were grouped on the CDR3 amino acid sequence using in‐house developed R scripts. In brief, the Hamming distance between clones was calculated. A dynamic threshold for the grouping of clones was used [12]. For every sample, a density function was calculated over the hamming distance matrix. This resulted in a bimodal distribution. The minimum between the two peaks was taken as a threshold with a minimum of three amino acids. Clones with a hamming distance below the dynamic threshold were grouped together to form a cluster and therefore considered to be related to each other.

## 3 Results

### 3.1 Patients

Three patients were included in this study, all diagnosed with CIDP variants [4, 5]. Paraproteins were absent in all three patients. At the time of diagnosis, testing for nodal/paranodal antibodies was not yet part of routine clinical care. Consequently, these data are unavailable for Patients B and C. In Patient A testing for nodal/paranodal antibodies was performed later for other research objectives, the results were negative.

*Patient A*: A 43‐year‐old woman initially presented with subacute hypesthesia in all limbs that developed within weeks, and was initially treated for Guillain‐Barré syndrome with a loading dose of intravenous immunoglobulins (IVIg) (2 g/kg over 5 days), which led to near‐complete symptom resolution. She experienced two subsequent relapses. The first relapse occurred 3 months after the initial treatment and was followed by a second IVIg loading dose. The second relapse occurred 9 weeks later, after which she was referred to our center. Upon referral, she presented with postural instability requiring a walking aid, lower limb hypesthesia and pain, while maintaining normal strength. At the time of the nerve biopsy, she had not received treatment with IVIg for 6 months. The diagnosis was revised to sensory‐dominant CIDP according to the EAN/PNS criteria [4] based on disease progression, demyelinating features on motor nerve conduction studies, elevated cerebrospinal fluid protein, and signs of demyelination/remyelination on electron microscopy. Long‐term IVIg treatment improved symptoms, including return of reflexes.

*Patient B*: A 76‐year‐old male presented with a gradual onset of predominantly asymmetric sensory symptoms, including pain. Initial considerations of vasculitis neuropathy were dismissed partly due to the results of the nerve biopsy. He met EFNS/PNS criteria for possible multifocal CIDP [5] based on probable conduction block in the left median nerve, nerve enlargement on ultrasound (not part of EFNS/PNS criteria at time of diagnosis), and nerve biopsy findings. Prior to the nerve biopsy the patient was untreated. The symptoms improved after 6 months of dexamethasone pulses, with minor residual sensory symptoms not necessitating additional treatment.

*Patient C*: A 39‐year‐old male initially presented with isolated sensory loss, notable for marked position and vibration deficits and areflexia. Initially, he did not meet EFNS/PNS criteria [5] for sensory CIDP due to absence of demyelinating features on motor nerve conduction studies and normal cerebrospinal fluid protein levels. Extensive genetic testing was negative. Nerve hypertrophy was observed on ultrasound. Prior to the nerve biopsy the patient was untreated. The symptoms did not improve with dexamethasone pulses but responded favorably to IVIg, resulting in improved coordination and gait stability. A relapse occurred in 2021 after IVIg withdrawal, manifesting with worsened coordination and distal weakness. Subsequent EMG confirmed motor nerve conduction criteria, leading to a diagnosis of typical CIDP according to current EAN/PNS criteria [5].

### 3.2 Pathological Examination

Histological examination of nerve tissue revealed findings consistent with a CIDP diagnosis in all three patients. Abnormalities primarily included focal loss of myelinated fibers on semi‐thin sections. However, more specific CIDP features, such as “onion bulbs” and reduced internodal lengths in teased nerve fibers, were not observed. A limited number of endoneural T‐cells were observed in all patients, with Patient A showing a small T‐cell infiltrate.

### 3.3 Highly Expanded TCR Clones Were Detected in Both Peripheral Blood and Nerve Biopsies of CIDP Patients

HECs were identified in peripheral blood samples from all three patients, consistent with our previous findings [6]. Most HECs were found at frequencies ranging from 0.5% to 2% of the TCRβ repertoire, with a single dominant clone representing up to 5% of the TCRβ repertoire in one patient (Patient B).

**FIGURE 1:**
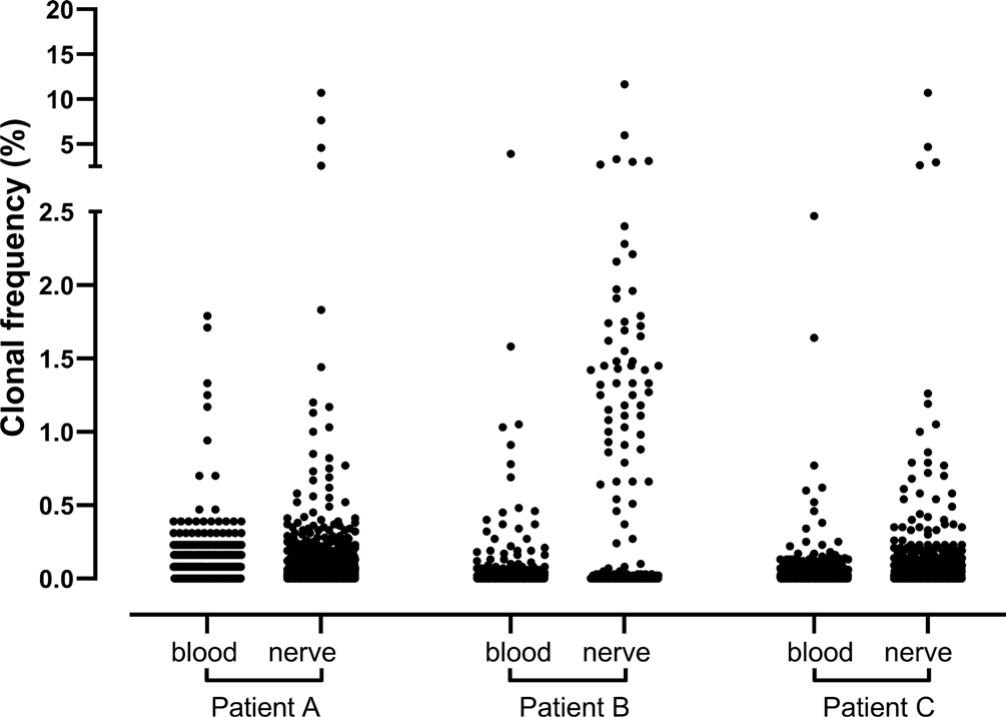
Scatter plot of the TCRβ repertoire in nerve tissues and peripheral blood of treatment‐naive CIDP patients. Each dot represents a unique TCRβ clone expressed as a percentage of the total TCRβ repertoire. TCRβ clones in blood with a frequency ≥ 0.5% of the total TCRβ repertoire are considered highly expanded clones (HECs). There are no pre‐defined cut‐off values for TCRβ clones in nerve tissue.

Furthermore, we detected clones in nerve tissue from all three patients, although with a more limited repertoire compared to peripheral blood. Notably, nerve tissue exhibited a higher frequency and cumulative impact of clones compared to those found in peripheral blood (Figure 1).

### 3.4 The Peripheral Blood TCRβ Repertoire Does Not Fully Reflect the TCRβ Repertoire Observed in Nerve Tissues

**FIGURE 2:**
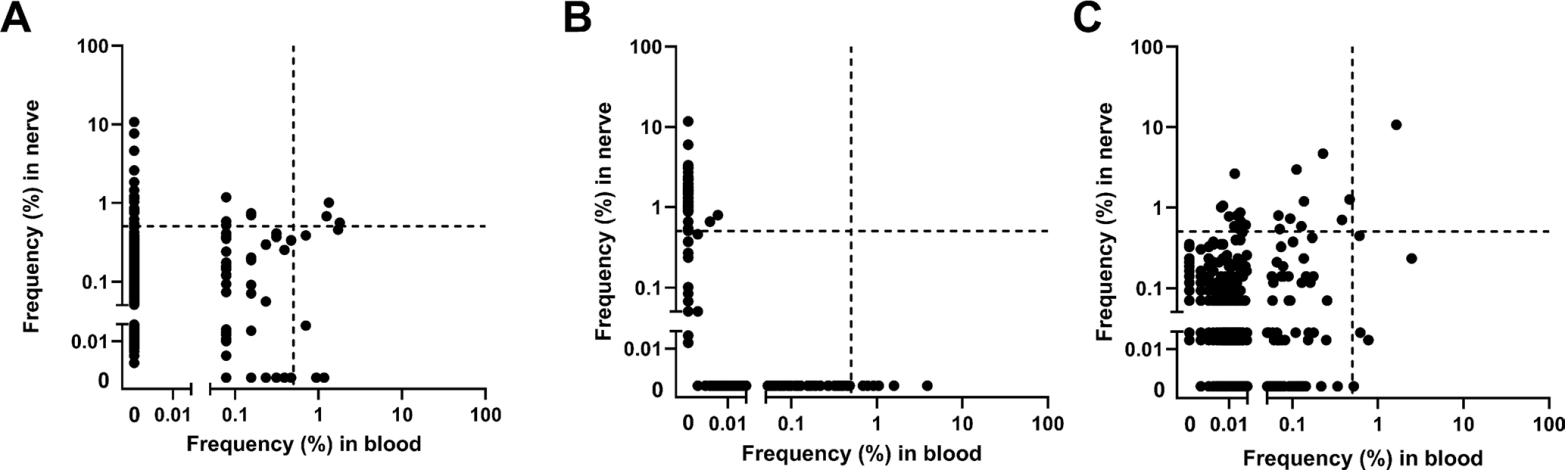
CDR3 clonal overlap plots between peripheral blood (X‐axis) and nerve tissue (Y‐axis) for (A) Patient A, (B) Patient B and (C) Patient C. Each dot represents a unique TCRβ clone, and its frequency in the analyzed repertoires is depicted on the x (peripheral blood) and y (nerve tissue) axes. The dotted lines indicate the 0.5% cut‐off for dominant TCRβ clones in blood.

We studied sharing of clones between peripheral blood and nerve tissue (Figure 2A–C). Nerve tissue‐restricted TCRβ clones were identified in all three patients. All patients showed high numbers of highly expanded TCRβ clones in nerve tissue that were not detected or detected only in very low frequencies in blood. In patients A and B the majority of these highly expanded clones were exclusively detected in nerve tissue (Figure 2A,B, top left corner).

Only a minor fraction of the shared clones was highly expanded in both nerve tissue and peripheral blood (Top right corner). In patient B almost no TCRβ clones were shared with the peripheral blood compartment, where the clonal frequency was very low (Figure 2B, bottom left corner). In patients A and C (Figure 2A–C) most of the HECs in peripheral blood were not highly expanded in nerve tissue.

Furthermore, we explored whether the nerve tissue‐restricted clones were newly formed or had any relationship with clones detected in blood. For each patient, we conducted a clustering analysis comparing the CDR3 amino acid sequences of all nerve tissue‐restricted clones (frequency ≥ 0.1%) with those from blood (frequency ≥ 0.1%). Our analysis revealed that in all three patients, nerve tissue‐restricted clones were distinct from blood clones, as evidenced by the absence of prominent clusters (Figure S1).

## 4 Discussion

In a previous study, we showed that while dominant T‐cell clones are present in peripheral blood samples of CIDP patients, this did not differ from healthy controls and the presence of these T‐cell clones did not correlate with treatment response or disease activity [6]. Following up on this study we performed TCRβ repertoire analysis on nerve tissue and blood in three CIDP patients with active disease. In the nerve tissue of all three patients we detected expanded TCRβ clones that were preferentially or uniquely identified in nerve tissue. In two of the three patients (A and C), some of the TCRβ clones were highly expanded both in the nerve tissue and in peripheral blood.

To shed more light on the relationship between nerve tissue T cells and peripheral blood T cells, we performed clustering analysis, which showed minimal clustering. This supports recent hypotheses that assume selective influx of T cells, locally‐induced proliferation and/or local differentiation of T cells in nerve tissue [13, 14].

In this hypothesis naïve T‐cells are thought to be activated in the lymphoid organs by antigen‐presenting cells presenting antigens derived from the myelin sheath, nerve tissue or mimicking components. Following activation, T‐cells undergo primary clonal expansion, proliferation, and differentiation into effector T‐cells. The activated effector cells exit the lymphoid organs and migrate into the bloodstream toward the nerve tissue, attracted by local homing signals such as adhesion molecules and chemokines, allowing T‐cells to cross the blood‐nerve barrier and infiltrate the nerve tissue. T‐cells with high‐affinity for local antigens will be selectively retained, and will undergo a second round of clonal expansion under the influence of (new) local antigens and high concentrations of inflammatory mediators. Due to a process called sequestration, the T‐cell clones are retained inside the nerve tissue, limiting their ability to migrate back into the circulation. Selective retention, local secondary clonal expansion, and sequestration thus may contribute to the unique T‐cell clonal profiles found in the nerve tissue of CIDP patients.

This sequence of events might explain why oral fingolimod, a disease‐modifying drug used in multiple sclerosis, was ineffective as a first‐line treatment in CIDP [13]. Fingolimod binds to sphingosine‐1‐phosphate (S1P) receptors on lymphocytes, causing them to remain in lymph nodes and preventing their migration into the circulation and to target tissues. Thus Fingolimod will not affect already circulating T‐cells nor the local expansion of T cells in nerve tissue.

Our study has some limitations. First, given that the indication for nerve biopsies is very limited, the number of patients in our study is limited. Also, these biopsies are only performed in selected patients and might not be representative for all patients with typical CIDP in whom nerve biopsies are not advised [4]. Furthermore, since CIDP is considered a heterogeneous disease, potential pathogenic highly expanded T‐cell clones in nerve tissue might only play a role in a subset of patients [4, 14], meaning that these results are possibly not generalizable to all patients.

Second, our analysis in blood does not capture the full TCR repertoire meaning that very low frequency clones might not be detected. Our sample of 2.5 mL of blood represents about ~0.05% of total blood volume, containing roughly 3 750 000 T cells. Even for rare naïve T‐cell clones, each TCRB sequence is found in 1 000–4 000 cells [15], while each cell on average expresses 21 TCRB mRNA molecules. We calculated that the chance of detecting at least one mRNA from a single cell is ~2.5%, increasing significantly with expanded clones (e.g., 22% for 10 cells, 92% for 100 cells). Therefore, dominant clones in both blood and nerve tissue should be detectable in our blood sample.

On a technical note a word of caution is needed regarding the results of patient B where 54 of the 56 highly expanded clones in the nerve tissue were unique for nerve tissue and not detectable in the peripheral blood. In this sample, only a limited number of CDR3s was identified (150 CDR3s among 5 928 reads), which could indeed be the result of selective retention, local secondary clonal expansion, and sequestration. However, it cannot be excluded that a low T cell number in the nerve tissue could have inflated the number of HECs in this patient. The latter might reflect disease heterogeneity regarding T‐cell influx in nerve tissue, or alternatively be the result of sampling error. Nonetheless, also in this patient the clones detected in nerve tissue are not detected or show an extremely low frequency in peripheral blood, supporting a nerve tissue specific TCR beta repertoire.

Finally, in this study we did not determine the phenotype, function or migrating properties of the HECs and therefore we can only speculate that these HECs in nerve tissue play a pathological role. Future studies could focus on determining the phenotype of the most expanded TCRβ clones in nerve tissue by sorting T‐cell subsets and perform reverse sequencing after sorting subsets.

## 5 Conclusion

The presence of unique T‐cell clones in the nerve tissue, not found in the blood, indicates a highly localized immune response with localized expansion and/or retention of T‐cells, driven by local antigens and the specific microenvironment within the nerve tissue.

The processes of selective retention, local secondary clonal expansion and sequestration, could contribute to the unique T‐cell HECs found in nerve tissue of CIDP patients. However, the findings of this study alone are insufficient to support this hypothesis. Future work tailored towards unravelling the phenotype and pathogenicity of these tissue restricted clones will be of importance.

## Conflicts of Interest

The authors declare no conflicts of interest.

## Supporting information


**Figure S1.** Network analysis/clustering plots. Each dot represents a unique CD3 sequence of a TCRβ clone with a frequency of ≥ 0.1%. The size of each dot represents the UMI count of each clone in the repertoire. All unique nerve tissue T‐cell clones (blue dots) were aligned to all clones present in blood (red dots). Up to three mismatches in the CD3 region were allowed. When there was a similarity of ≤ 3 amino acids between CD3 sequences, the sequences were considered to be related. A relationship is visualized as a connecting line between the dots in the figure.

## Data Availability

The data that support the findings of this study are available from the corresponding author upon reasonable request.
