# Supplementary material for: Unique Nerve Tissue‐Restricted T‐Cell Clones in Chronic Inflammatory Demyelinating Polyneuropathy
Source: J Peripher Nerv Syst. 2025 Feb 18;30(1):e70006. doi: 10.1111/jns.70006 (PMC11836545; doi:10.1111/jns.70006)
Supplement: Supplementary file 1 — Figure S1. Network analysis/clustering plots. Each dot represents a unique CD3 sequence of a TCRβ clone with a frequency of ≥ 0.1%. The size of each dot represents the UMI count of each clone in the repertoire. All unique nerve tissue T‐cell clones (blue dots) were aligned to all clones present in blood (red dots). Up to three mismatches in the CD3 region were allowed. When there was a similarity of ≤ 3 amino acids between CD3 sequences, the sequences were considered to be related. A relationship is visualized as a connecting line between the dots in the figure. [file JNS-30-0-s001.docx]

**Supplementary data**

Patient A


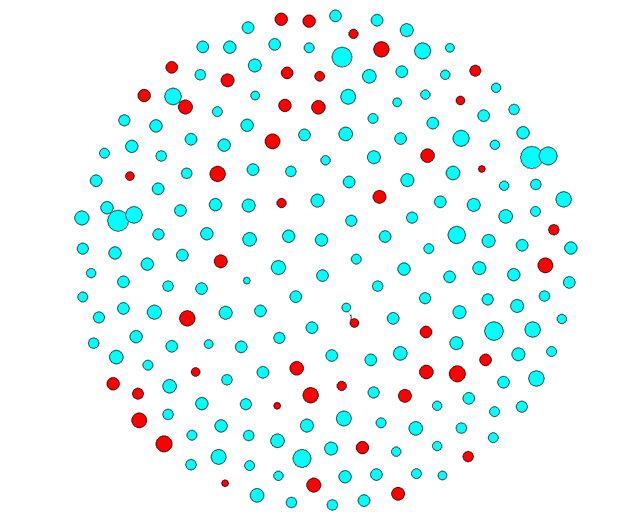


Patient B


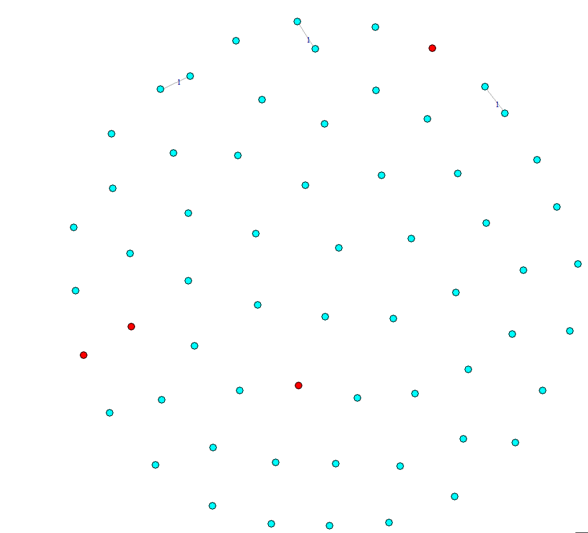


Patient C


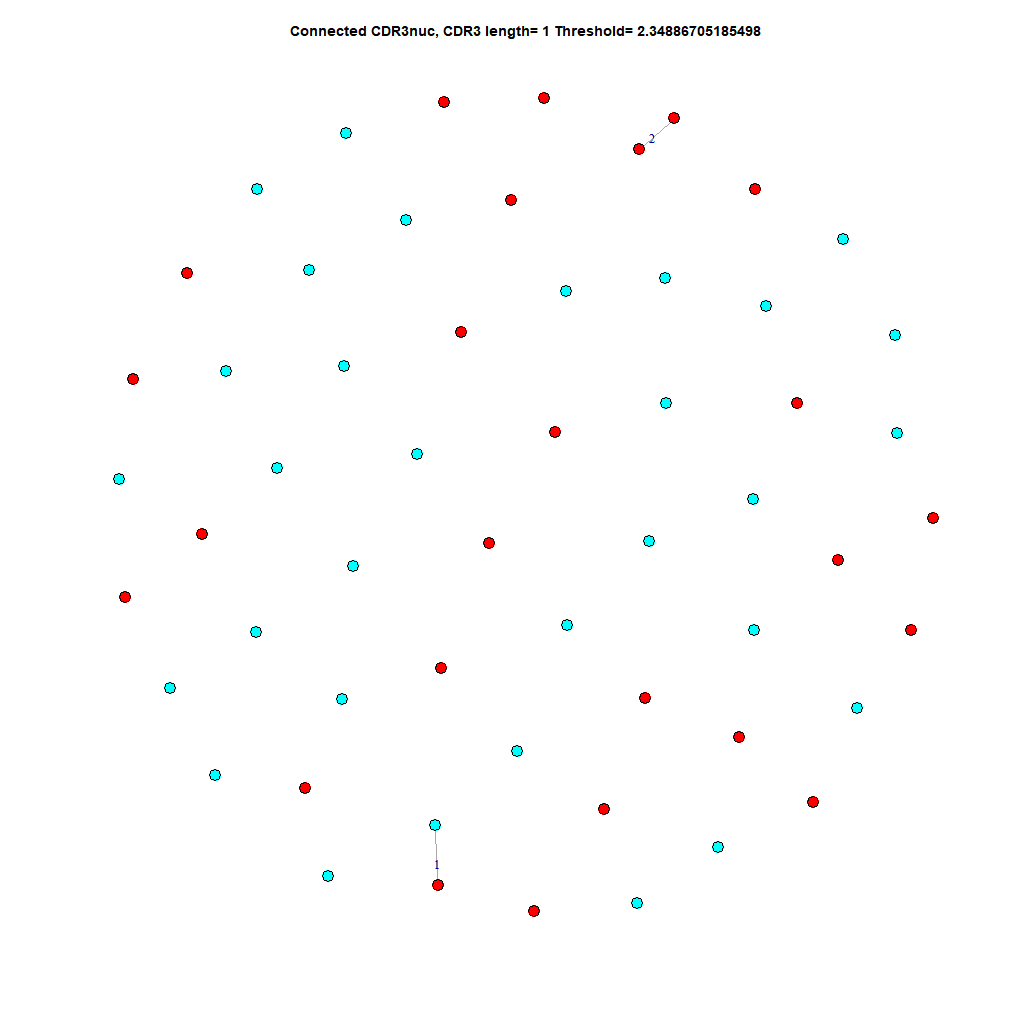


**Supplementary figure 1: Network analysis/clustering plots.**Each dot represents a unique CD3 sequence of a TCRβ clone with a frequency of ≥ 0.1%. The size of each dot represents the UMI count of each clone in the repertoire. All unique nerve tissue T-cell clones (blue dots) were aligned to all clones present in blood (red dots). Up to three mismatches in the CD3 region were allowed. When there was a similarity of ≤ 3 amino acids between CD3 sequences, the sequences were considered to be related. A relationship is visualized as a connecting line between the dots in the figure.
